# Aurora-A Induces Chemoresistance Through Activation of the AKT/mTOR Pathway in Endometrial Cancer

**DOI:** 10.3389/fonc.2019.00422

**Published:** 2019-05-22

**Authors:** Jun Wu, Ziyun Cheng, Xiaofeng Xu, Jian Fu, Kaiyue Wang, Tao Liu, Chan Wu, Xiangyi Kong, Qian Yang, Guijun Yan, Huaijun Zhou

**Affiliations:** ^1^Department of Gynecology, The Affiliated Drum Tower Hospital of Nanjing University Medical School, Nanjing, China; ^2^Department of Gynecology and Obstetrics, The First Affiliated Hospital of Xi'an Jiaotong University, Xi'an, China; ^3^Suqian People's Hospital of Nanjing Drum Tower Hospital Group, Department of Gynecology, Suqian, China; ^4^Department of Gynecology, The First People Hospital of Changzhou, Changzhou, China; ^5^Department of Gynecology and Obstetrics, The Nanjing Pukou Hospital, The Fourth Affiliated Hospital of Nanjing Medical University, Nanjing, China; ^6^Reproductive Medicine Center, The Affiliated Drum Tower Hospital of Nanjing University Medical School, Nanjing, China

**Keywords:** AKT signaling, Aurora-A, Chemoresistance, Endometrial cancer, mTOR signaling

## Abstract

Endometrial cancer (EC) is the most common gynecological tumor all over the world, and advanced/metastatic EC remains a malignancy with poor survival outcome due to highly resistant to conventional chemotherapeutic treatment. Here, we report that Aurora-A, a serine-threonine kinase, plays a vital role in chemoresistance of EC. Aurora-A is overexpressed in EC tissues, compared with normal endometrium and Aurora-A expression is associated with decreased overall survival. Overexpression of Aurora-A in EC cell lines (Ishikawa and HEC-1B cells) promotes cell proliferation and induced paclitaxel- and cisplatin-resistance. Furthermore, Aurora-A activating AKT-mTOR pathway further induces chemoresistance *in vitro*, consistent with a positive correlation between Aurora-A and phosphorylated AKT/4E-BP1 expression in EC tissues. In summary, our study provides the strong evidence that Aurora-A controls the sensitivity of EC cell lines to chemotherapy via AKT/mTOR pathway, indicating that pharmacologic intervention of Aurora-A and AKT/mTOR in combination with chemotherapy may be considered for the targeted therapy against EC with overexpression of Aurora-A.

## Introduction

Endometrial cancer (EC) is the most common gynecological cancer, and its incidence is increasing (1). In 2012, it occurred in 320,000 women and caused 76,000 deaths worldwide (2), which is placing a heavy burden on individuals, families, and society. The current optimal approach for EC treatment is surgical resection with adjuvant chemotherapy or radiotherapy. Although early-stage EC has a favorable prognosis, the advanced or recurrent EC has a poor prognosis because of chemoresistance (3). Therefore, the molecular mechanisms promoting EC progression need to be further studied and a strategy to overcome and/or prevent chemoresistance is crucial to improve efficacy of EC treatment.

Aurora-A, a serine-threonine kinase, is reported to induce centrosome amplification, aneuploidy, and transformation in mammalian cells (4). Aberrant expression of Aurora-A has been implicated in the initiation, development, and progression of a wide range of malignancies, including colon carcinoma, lymphoma, gastrointestinal adenocarcinomas, breast cancer, and bladder cancer (5–7). As soon as a strong link between Aurora-A and cancer was found, many pharmaceutical companies quickly started trying to develop Aurora-A inhibitors for cancer treatment. Inhibitors of Aurora kinases, such as MLN8237 (8), MK-5108 (9), and ENMD-2076 (10), have been developed but none have yet gone beyond Phase III trials (5), suggesting the functions of Aurora-A have not been fully understood in cancer cells. More importantly, in a complex organism, Aurora-A is an oncogene in mammary epithelium and glands (11, 12), whereas it could be a tumor suppressor in neural stem cells (13), so it should be considered that Aurora-A has cell type-specific functions.

Due to its role as a spindle checkpoint regulator, Aurora-A has been implicated in conferring resistance to chemotherapy in cancer cells. Increasing evidences have shown that overexpression of Aurora-A is associated with chemoresistance (14–19). Recent reports showed that Aurora-A is correlated with the resistance to carboplatin/Cisplatin and indicates a poor prognosis (20, 21). Aurora-A has been noted to be a novel therapeutic target for the gynecological malignancies, however, only a few reports have described a role for Aurora-A in EC. Recent study showed a correlation between overexpression of Aurora-A and clinicopathological factors, and Aurora-A increased chemosensitivity to paclitaxel (22). Aurora-A induces chemoresistance through ERK and mTOR pathways in breast cancer cells (23), and activates Akt via a p53-dependent manner in ovarian cancer cells(24), To date, the underlying molecular mechanism of Aurora-A-mediated chemoresistance in EC is unclear.

In the present study, we reveal the underlying mechanism of Aurora-A-mediated chemoresistance in EC. Aurora-A is essential to promote cell proliferation and enhance cell resistance to cisplatin and paclitaxel by activation of AKT/mTOR signaling pathway *in vitro*, consistent with a positive correlation between Aurora-A and phosphorylated AKT/4E-BP1 expression in EC tissues. These findings indicate that pharmacologic intervention of Aurora-A and AKT/mTOR in combination with conventional chemotherapy is an attractive therapeutic approach for EC treatment.

## Methods

### Clinical Samples

Tissue samples were collected from 90 cases of endometrial cancer paraffin-embedded specimens, which were pathologically and clinically diagnosed as EC at the Affiliated Drum Tower Hospital of Nanjing University Medical School from 2007 to 2017. In addition, 12 cases of freshly normal proliferative tissue endometrium and 23 cases of EC tissues were frozen and stored for RNA extraction and western blot analysis. Patients with other diseases or patients who underwent special therapies before surgery were excluded. The samples were provided by the Tissue Bank of the Affiliated Drum Tower Hospital of Nanjing University Medical School. The age of the participants was range from 42 to 68 years. A written form of consent was obtained from each participant prior to the surgery, and this study was approved by the medical ethics committee of the Affiliated Drum Tower Hospital of Nanjing University Medical School.

### Clinicopathological Characterization of Clinical EC Specimens

RNA-Seq gene expression HTSeq-Counts data for the TCGA-CESC Project was downloaded from TCGA official website (https://cancergenome.nih.gov).The corresponding clinical information was downloaded from cBioportal (http://www.cbioportal.org). The relationship between Aurora-A and clinical-pathologic features was analyzed with the Wilcoxon signed-rank test and logistic regression. Kaplan–Meier survival curves were created using the log-rank test for TCGA database to compare high and low levels of Aurora-A groups.

### Immunohistochemistry (IHC) Analysis

IHC analysis of Aurora-A and other proteins expressed in paraffin sections of tissues from EC patients were carried out as described previously (25). Antibodies used in this study were Rabbit monoclonal antibody against Aurora-A (Cell Signaling Technology, #91590, 1:250 dilution), mouse monoclonal antibody against Phospho-Akt (Ser473) (Cell Signaling Technology, #4051, 1:200 dilution) and rabbit monoclonal antibody against Phospho-4E-BP1 (Thr37/46) (Cell Signaling Technology, #2855, 1:1600 dilution). The stained sections were evaluated using an Olympus microscope. The IHC-stained sections were reviewed and scored independently by two experienced pathologists who had no knowledge of the patients' identities or clinical status, and both pathologists had similar accuracy rates. Protein expressions were scored semi-quantitatively by a manual histo-score (H-score) methodology based on staining intensity and percentage of positive tumor cells. Strongly staining scored 3, moderately staining scored 2, weakly staining scored 1, and negatively staining scored 0. The H-score of protein expression is obtained by the formula 3 x percentage of strongly staining + 2 x percentage of moderately staining + percentage of weakly staining, giving a range of 0 ± 300.

### Quantitative Real-Time PCR

Total RNA from cell lines and tissue samples was extracted using TRIzol reagent (Invitrogen). cDNA was reverse-transcribed from 1 μg total RNA with HiScript II 1st Strand cDNA Synthesis Kit (Vazyme). Real-time RT-PCR was conducted using a ChamQ Universal SYBR qPCR Master Mix (Vazyme) with the following primer pairs: *Aurora-A*, 5′- TGGAATATGCACCACTTGGA−3′ and 5′- ACTGACCACCCAAAATCTGC−3′; and *GAPDH*, 5′- GAGTCAACGGATTTGGTCGT−3′, and 5′- TTGATTTTGGAGGGATCTCG−3′. Samples were run in duplicate using RNA preparations from three independent experiments. The fold change in the expression of each gene was normalized to the expression of the internal control (*GAPDH*), and the data were analyzed using the 2^−ΔΔ*CT*^ method.

### Western Blot Analysis

Western blot analysis was performed as described previously (26). Rabbit monoclonal antibody against Aurora-A (Cell Signaling Technology, #91590, 1:1000 dilution), rabbit monoclonal antibody against Akt (pan) (Cell Signaling Technology, #4685, 1:2000 dilution), mouse monoclonal antibody against Phospho-Akt (Ser473) (Cell Signaling Technology, #4051, 1:2000 dilution), rabbit monoclonal antibody against 4E-BP1 (Cell Signaling Technology, #9644, 1:2000 dilution), rabbit monoclonal antibody against Phospho-4E-BP1 (Thr37/46) (Cell Signaling Technology, #2855, 1:500 dilution), and mouse monoclonal antibody against GAPDH (Proteintech, 1E6D9, 1:10,000 dilution) were used.

### Cell Cultures and Transfection

The HEK 293T cells, the human endometrial cancer cell lines, Ishikawa and HEC-1B cells were purchased from the American Type Culture Collection. These cells were cultured in Dulbecco's modified minimum essential medium containing 10% fetal bovine serum (Gibco) and 1% penicillin/streptomycin (HyClone Laboratories) under standard incubation conditions (37°C, 5% CO_2_). For transient transfection, HEC-1B cells were transfected with 0.5, 1, or 2 μg of Aurora-A plasmids (normalized to 2 μg with vector) using Lipofectamine 3,000 (Invitrogen).

### Virus Construct

To generate the *Aurora-A* lentiviral construct, the open reading frame of human *Aurora-A* was amplified from Ishikawa cells cDNA by PCR using high fidelity polymerase (Prime STAR® HS DNA Polymerase, TaKaRa) and was subcloned into the pCDH-CMV-MCS-EF1-copGFP vector (System Biosciences). The vector is a lentivector expression system, which contained a lentiviral vector carrying the green fluorescent protein (GFP) gene. Finally, the plasmid construct (pCDH-CMV- EF1- Aurora-A -copGFP) was verified by sequencing. The empty pCDH-CMV-MCS-EF1-copGFP vector expressed only GFP as the control. shRNA expressing lentiviral constructs against human AKT and mTOR from the RNAi consortium human collection were purchased from Sigma.

### Lentivirus Generation and Infection

Lentivirus was prepared according to the manufacturer's instructions (System Bioscience). Briefly, a lentiviral plasmid was co-transfected with pLP1, pLP2, and VSVG40 into 293T cells by Lipofectamine 3000 (Invitrogen). The medium containing viral particles were collected 48 and 72 h after transfection. The supernatants were collected and filtered through a 0.45 μm filter and stored at −80°C until further use. Where appropriate, the packaged viruses were tittered by infection of Ishikawa cells and HEC-1B cells, and FACS analysis of GFP-positive cells 48 h after Aurora-A infection.

### Cell Proliferation Assay

Cell proliferation was evaluated using the Cell Counting Kit-8 (CCK-8) (Vazyme) according to the manufacturer's instructions. Ishikawa and HEC-1B cells were seeded on to a 96 well plate in triplicate at a concentration of 1 × 10^3^ cells/ml. Cells were incubated and periodically analyzed using the CCK-8. Readings at 450 nm were obtained at 24, 48, 72, 96, and 120 h. Each assay was repeated in triplicate, and an increase in absorbance indicated an increase in the cell number.

### Cell Viability Assay

Cell viability was evaluated using the Cell Counting Kit-8 (CCK-8) (Vazyme) according to the manufacturer's instructions. Ishikawa and HEC-1B cells were seeded in 96-well plates (2× 10^3^ cells/ well), respectively. Cells in each well were incubated with 10 μl of CCK-8 diluted in normal culture medium at 37°C for 2 h. Survival rates were determined at 24, 48, and 72 h after transfection with OD measurements at 450 nm.

### Pharmacologic Inhibition

Ishikawa and HEC-1B cells were treated with 2.5 μM Perifosine (Selleck) or 200 nM RAD001 (Selleck) dissolved in DMSO. To determine the chemosensitivity of EC Ishikawa and HEC-1B cell lines, cells were treated with increasing concentrations (generally 0–64 μM) of paclitaxel (Sigma) and Cisplatin (Sigma) for cell viability assay at 48 h. The concentration of each drug killing half of cells was selected, respectively. All experiments using chemical treatments used vehicle DMSO treatment as the control.

### Gene Set Enrichment Analysis

The gene expression data were downloaded from TCGA official website. GSEA (Gene Set Enrichment Analysis) is a computational method that determines whether an a priori defined set of genes shows statistically significant, concordant differences between two biological states (27). In this study, GSEA firstly generated an ordered list of all genes according to their correlation with Aurora-A expression, GSEA was carried out to elucidate the significant survival difference observed between high- and low- Aurora-A groups. Gene set permutations were performed 1,000 times for each analysis. The expression level of Aurora-A was used as a phenotype label. The nominal *P*-value and the normalized enrichment score (NES) were used to sort the pathways enriched in each phenotype.

### Statistical Analysis

All statistical analysis was performed using SPSS 22.0 software and GraphPad Prism 6. For the analysis of the correlation between Aurora-A expression and clinicopathologic features of EC patients, Pearson's χ*2* test was used. Multivariate Cox analysis was used to compare the influence of Aurora-A expression on survival along with other clinical characteristics. For comparisons of two groups of normally distributed data, unpaired two-tailed Student's *t*-test was used. For multiple comparisons, one-way analysis of variance (ANOVA) followed by *post hoc Tukey's* test was performed. Statistical results were presented as means ± standard error of the mean (S.E.M.). Asterisks indicate critical levels of significance (^*^P < 0.05, ^**^P < 0.01, and ^***^P < 0.001).

## Results

### Aurora-A Expression Levels Increase in Human EC Tissues Compared With Normal Endometrium Tissues

**Figure 1:**
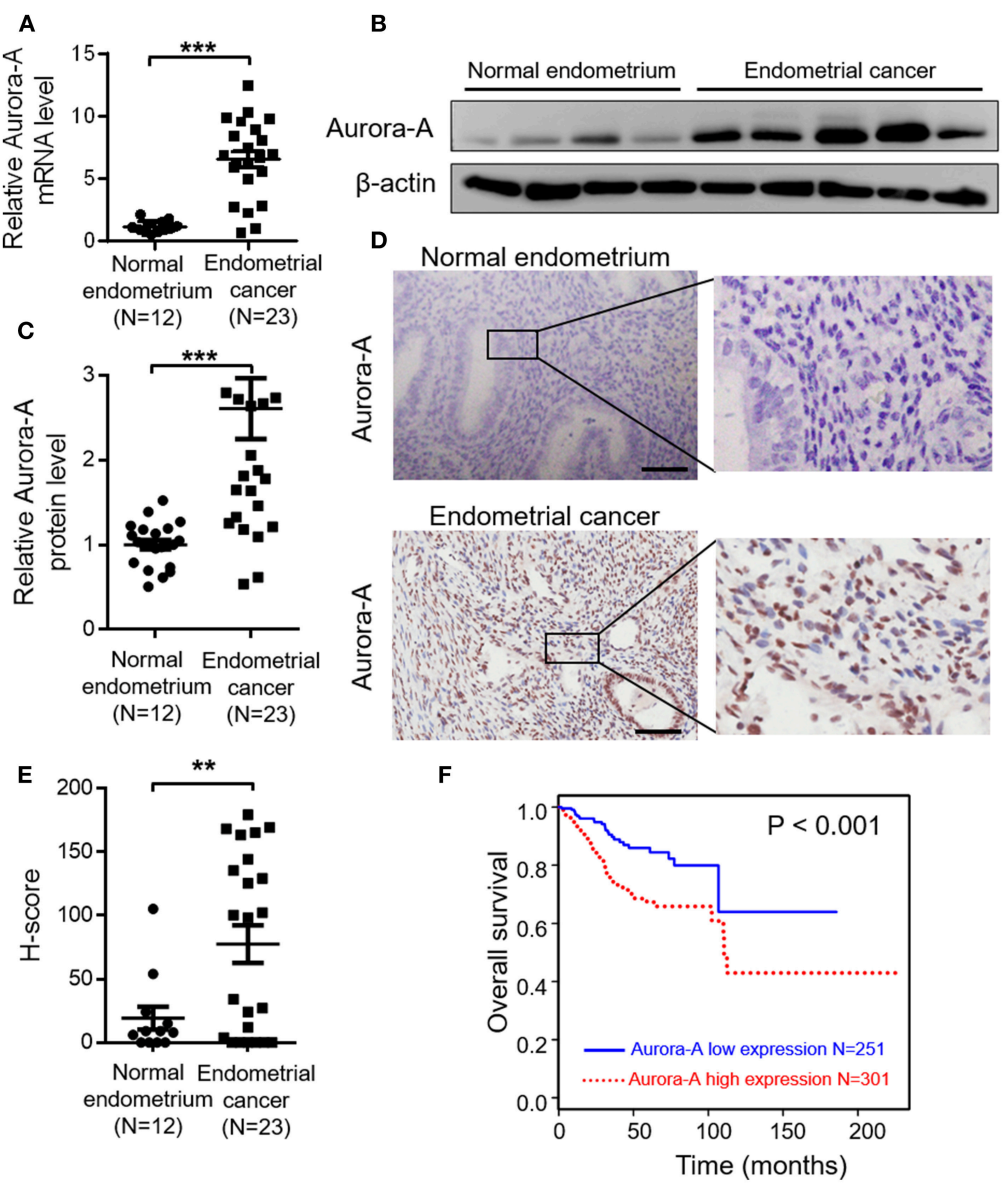
Aurora-A overexpression correlates with poor prognosis of endometrial cancer. **(A)** Real-time quantitative PCR showing increased *Aurora-A* mRNA expression in EC tissues compared with normal endometrium. *GAPDH* is used as an internal control. Data are expressed as means ± S.E.M., Student's *t*-test, ***P < 0.001. **(B)** Representative western blots showing increased Aurora-A protein level in EC tissues compared with normal endometrium. β-actin is used as loading control. **(C)** Quantification of relative Aurora-A level. The images of western blots were quantified using Image J software. Data are expressed as means ± S.E.M., Student's *t*-test, ***P < 0.001. **(D)** Representative IHC staining showing Aurora-A expression. Scale bar, 100 μm. **(E)** H-Scores of Aurora-A levels were presented as scatter diagram in **(D)**. Data are expressed as means ± S.E.M., Student's *t*-test, **P < 0.01. **(F)** Kaplan–Meier curves are made to show the survival rate of 552 EC patients with high and low *Aurora-A* mRNA expression levels. The cut-off value of Aurora-A expression is 7.22.

*Aurora-A* mRNA expression in 23 human EC tissues and 12 normal endometrium was detected by quantitative real time-PCR (qRT-PCR) analysis, and the result showed that EC tissues exhibit significantly increased *Aurora*-*A* transcript level, compared with normal endometrium (*P* < 0.001) (Figure 1A). Moreover, Aurora-A protein expression in these tissues was analyzed by western blot. Immunoblot analysis revealed a much higher expression of Aurora-A in EC tissues compared with normal tissues (*P* < 0.001) (Figures 1B,C). Further immunohistochemistry (IHC) analysis showed that Aurora-A expression was barely detectable in normal endometrium, whereas a strong signal was detected in EC tissues (Figures 1D,E). Interestingly, the cytoplasmic protein Aurora-A was mainly located in the nucleus of EC cells. Therefore, the above data provided strong evidence that the expression levels of Aurora-A in human EC tissues were higher than that of normal endometrium.

### Overexpression of Aurora-A Is Correlated With Poor Prognosis of EC Patients

Having demonstrated that Aurora-A expression increases in EC, we next examined the relationship between Aurora-A gene expression and patient survival in 552 cases from TCGA database. Best expression cut-off value of Aurora-A is 7.22. Kaplan-Meier survival analysis showed that the survival rate was significantly lower in tissues with high Aurora-A expression compared with tissues with low Aurora-A expression (Figure 1F). Univariate Aurora-A expression as a categorical dependent variable was associated with poor prognostic clinicopathologic characteristics (Table 1A). Significant risk factors (*p* < 0.05) in the univariate analysis were entered into the multivariate analysis using the logistic regression model. At multivariate analysis, Aurora-A remained independently associated with overall survival, with a HR of 1.665 (CI: 1.026–2.7031.25–12.64, *p* = 0.039), along with stage (Table 1B). This finding suggested that Aurora-A expression is associated with poor prognosis in human EC.

**Table 1:** **(A)** Overall survival and associations with clinicopathologic characteristics.

**Clinicopathologic variable**	**Hazard ratio (95%CI)**	***P*-value**
**(A**)		
Age (≤50 vs. >50)	2.016 (0.739–5.503)	0.171
Aurora A expression (high vs. low)	2.408 (1.517–3.821)	< 0.001
BMI (≤30 vs. >30)	1.049 (0.684–1.609)	0.826
Grade (G1, G2, vs. G3)	3.227 (1.872–5.562)	< 0.001
Status (tumor free vs. with tumor)	8.412 (5.479–12.915)	< 0.001
Histology (EA vs. Non-EA)	0.346 (0.228–0.523)	< 0.001
Stage (I, II vs. III, IV)	3.943 (2.601–5.977)	< 0.001
Surgical approach (Open vs. Mini invasive)	1.328 (0.862–2.047)	0.198
Myometrial invasion (≤50 vs. >50%)	3.662 (2.230–5.780)	< 0.001
Pelvic peritoneal cytology (positive vs. negative)	4.650 (2.840–7.616)	< 0.001
Pelvic lymph nodes (positive vs. negative)	4.312 (2.644–7.034)	< 0.001
Para-aortic lymph nodes (positive vs. negative)	3.528 (1.892–6.577)	< 0.001
**(B)**		
Aurora A expression (high vs. low)	1.665 (1.026–2.703)	0.039
Stage (I, II vs. III, IV)	3.489 (2.238–5.439)	< 0.001

***(B)** Multivariate survival model after variable selection. BMI, Body mass index; EA, Endometrioid adenocarcinoma*.

### Aurora-A Promotes Cell Proliferation and Induces Paclitaxel- and Cisplatin-Resistance in Human EC Cell Lines

**Figure 2:**
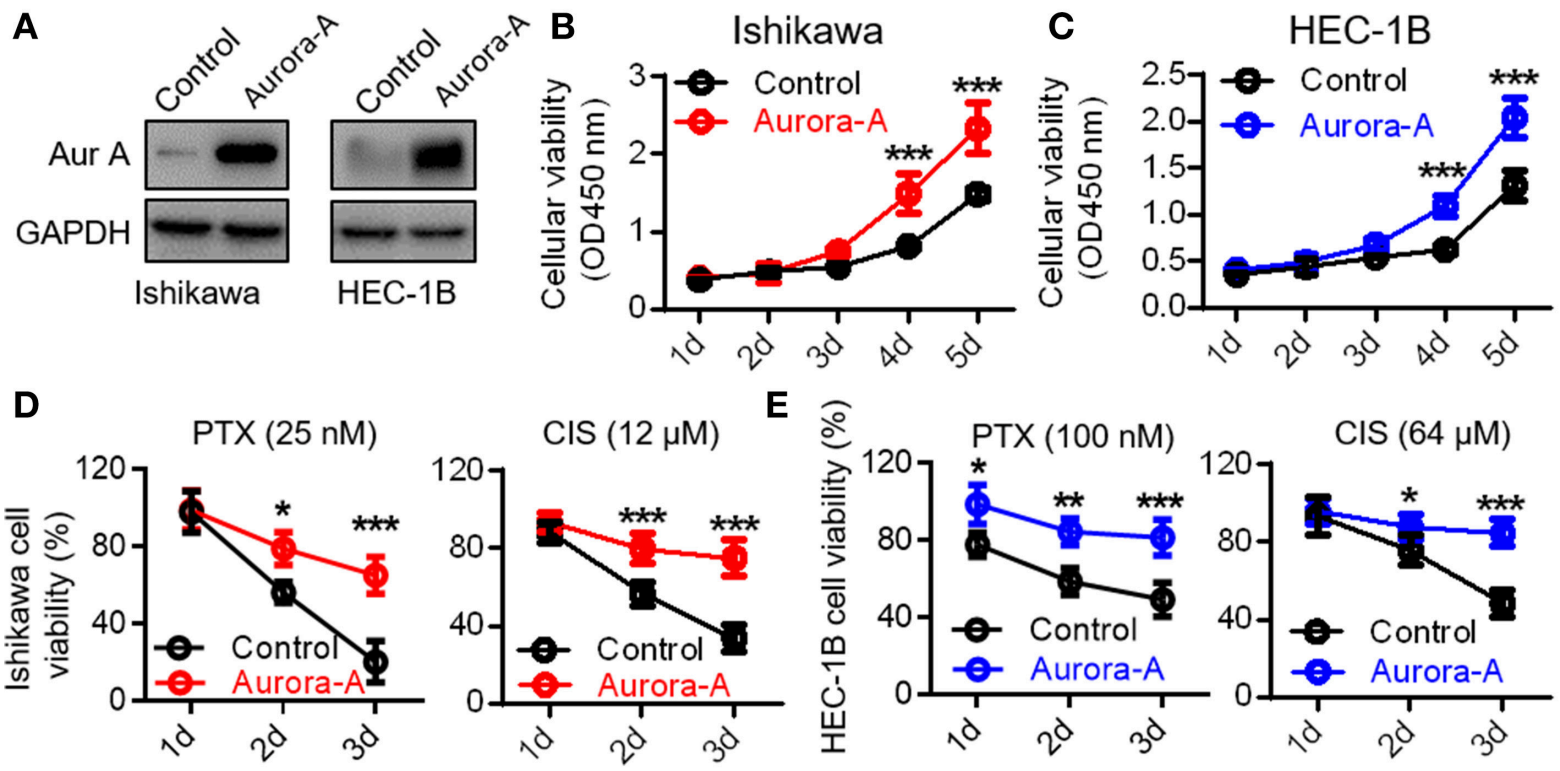
Aurora-A promotes cell proliferation and induces paclitaxel- and cisplatin-resistance in Ishikawa and HEC-1B cell lines. **(A)** Western blotting confirming Aurora-A protein level in Aurora-A-stable expressing cell lines compared with control cells. GAPDH is used as loading control. **(B,C)** A CCK-8 assay is performed to show overexpressed Aurora-A promotes cell proliferation in Ishikawa and HEC-1B cell lines. **(D)** A CCK-8 assay is performed to evaluate cell viability of PTX- and CIS-resistance in Ishikawa cells. PTX, paclitaxel; CIS, cisplatin. **(E)** A CCK-8 assay is performed to evaluate cell viability of PTX- and CIS-resistance in HEC-1B cells. Data are expressed as means ± S.E.M., one-way ANOVA, *N* = 5, ***P < 0.001, **P < 0.01, and *P < 0.05 in **(B–E)**.

To investigate the physiological function of Aurora-A, we established two Aurora-A stable-expressing EC cell lines (Ishikawa and HEC-1B) using lentiviral expression system. Western blot analysis confirmed that these cells with markedly increased Aurora-A level, compared to cells transfected with empty vector (Figure 2A). These cells were subsequently subjected to CCK8 assay. The results showed a significant increase of the cell proliferation index over a period of 5 days in Ishikawa cells of overexpressing Aurora-A (*P* < 0.001) (Figure 2B); a similar phenotype was observed in HEC-1B cells (Figure 2C), suggesting a promotion role of Aurora-A in human EC cells proliferation. To investigate whether Aurora-A induces chemoresistance, we evaluated the cell viability of EC cells treated with paclitaxel (PTX) and cisplatin (CIS), both of which are commonly used anticancer drugs in EC. We optimized the concentrations (for Ishikawa cell, PTX: 25 nM; CIS: 12 μM; for HEC-IB cell, PTX: 100 nM; CIS: 64 μM) for chemoresistance(Supplementary Figure 1) and evaluated the PTX and CIS effects on Ishikawa and HEC-IB cell viability. The results showed that Aurora-A dramatically confers the two cells resistant to PTX and CIS in comparison with the control (Figures 2D,E).

### Aurora-A Activates AKT-mTOR Pathway *in vitro*

**Figure 3:**
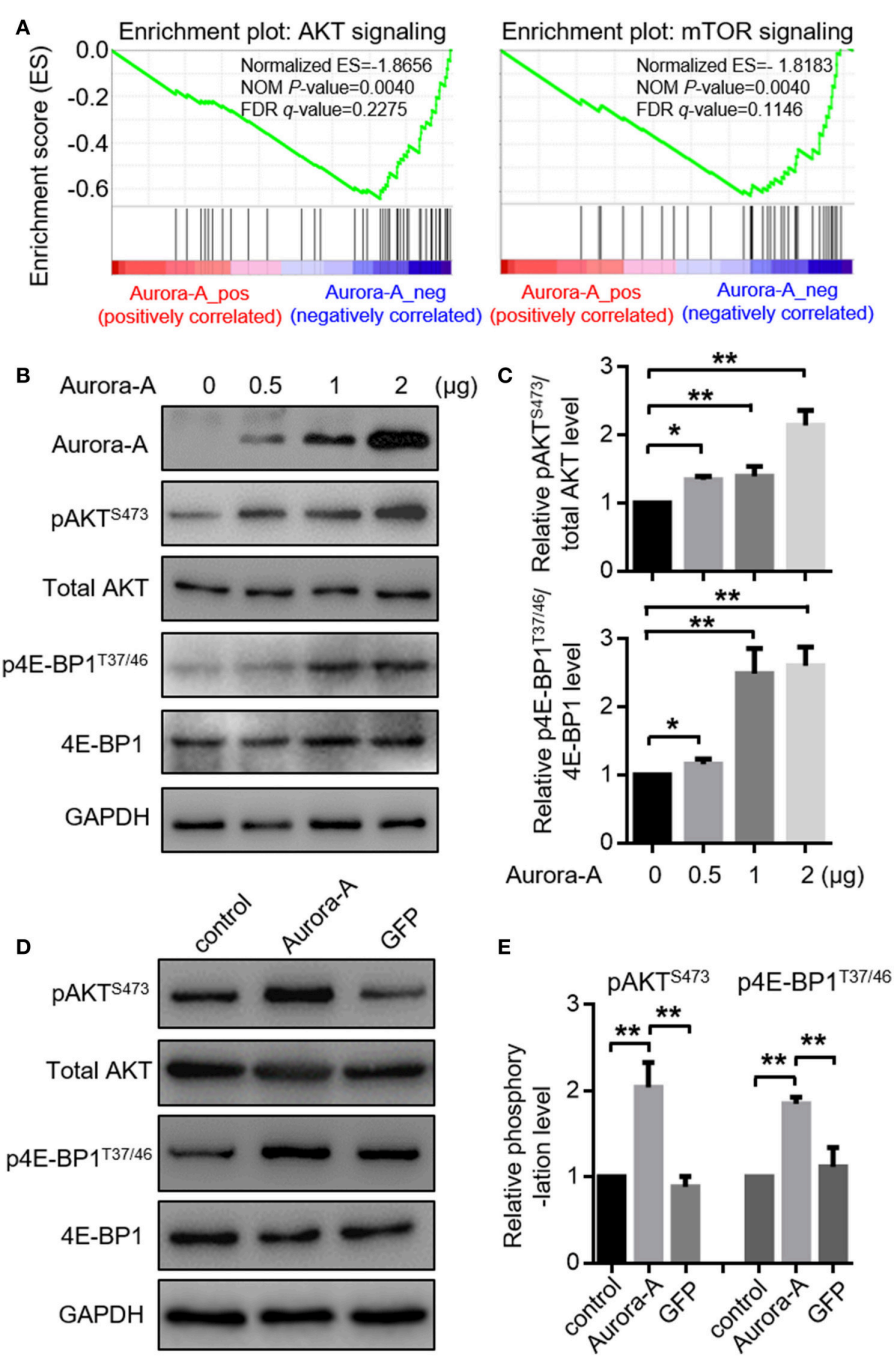
Aurora-A activates AKT and mTOR pathways *in vitro***. (A)** Gene expression data acquired from TCGA database are subjected to GSEA for analysis, which strongly indicated that Aurora-A may be associated with AKT and mTOR signaling pathways. NOM P-Val, normalized *p*-value. FDR q-Val: false discovery rate q-value. **(B)** Aurora-A increased AKT and mTOR signaling pathway. Representative blots of Aurora-A, pAKT^S473^, total AKT, 4E-BP1, and p4E-BP1^T37/46^ levels in HEC-1B cells transiently transfected with 0.5, 1, or 2 μg of Aurora-A plasmids (normalized to 2 μg with vector).**(C)** Quantification of pAKT^S473^/AKT and p4E-BP1^T37/46^/4E-BP1 ratio fold change (normalized) observed in **(B)**. Data are expressed as means ± S.E.M., Student's *t*-test, *N* = 3, **P < 0.01 and *P < 0.05. **(D)** Aurora-A activated AKT and mTOR signaling pathway. Representative blots of pAKT^S473^, total AKT, 4E-BP1, and p4E-BP1^T37/46^ levels in control cells, Aurora-A-overexpressing cells and GFP-overexpressing cells. GFP was used as the control. **(E)** Quantification of pAKT^S473^/AKT and p4E-BP1^T37/46^/4E-BP1 ratio fold change (normalized) observed in **(E)**. Data are expressed as means ± S.E.M., Student's *t*-test, *N* = 3, **P < 0.01.

To investigate the details molecular mechanisms underlying Aurora-A-induced chemoresistance in EC, we conducted Gene Set Enrichment Analysis (GSEA) between low and high Aurora-A expression data sets to predict the signaling pathways potentially involved. GSEA result revealed significant differences (FDR < 0.05, NOM *P* < 0.05) in enrichment of MSigDB Collection (c2.cp.biocarta and h.all. v6.1. symbols). We then selected the most significantly enriched signaling pathways based on their normalized enrichment score (NES) (Supplementary Table 1). The Figure 3A showed that are AKT and mTOR signaling were enriched in Aurora-A high expression phenotype, strongly indicating that the two signaling may be involved in EC development.

We thus examined AKT pathway recruitment by overexpressing of Aurora-A using western blot analysis of phospho-AKT^S473^ (pAKT^S473^); HEC-1B cells were transfected with 0.5, 1, or 2 μg of Aurora-A plasmids, the cells exhibited increased pAKT^S473^ levels in a dose-dependent manner (P < 0.001) (Figure 3B,C). AKT canonically regulates mammalian target of rapamycin (mTOR), thus we examined the effect of Aurora-A expression on mTOR activity via detecting the level of phospho-4E-BP1^T37/46^ (p4E-BP1^T37/46^), revealing an increment of p4E-BP1^T37/46^ relative to total 4E-BP1 following Aurora-A expression (Figures 3B,C). Moreover, both pAKT^S473^ and p4E-BP1^T37/46^ levels were significantly increased in Aurora-A stable-expressing HEC-1B cells (*P* < 0.001) (Figures 3D,E), further confirmed that Aurora-A can activate AKT-mTOR pathway in HEC-1B cells.

### Aurora-A Induces Chemoresistance by Activation of the AKT-mTOR Pathway in EC Cell Lines

**Figure 4:**
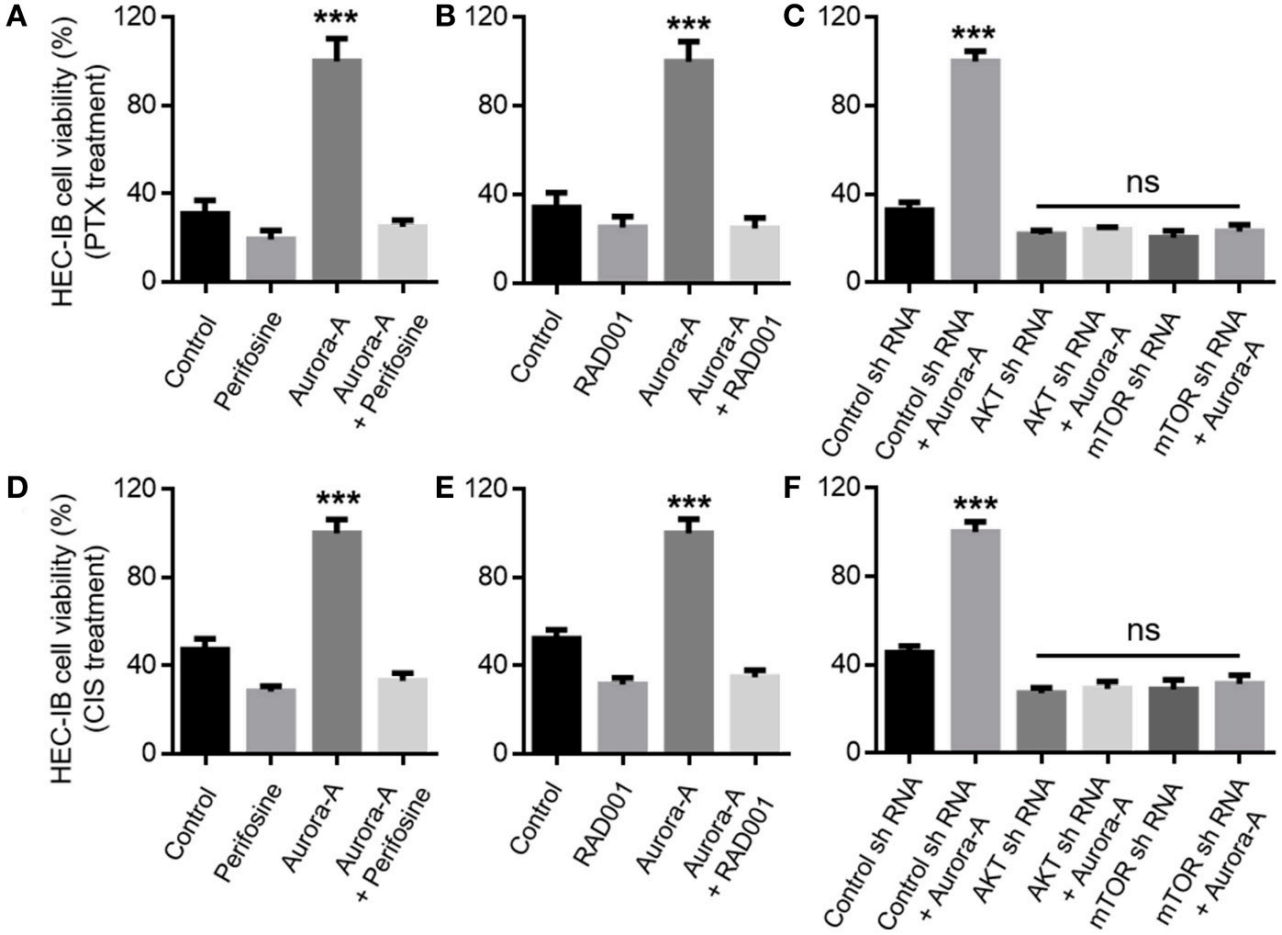
Aurora-A induces chemoresistance by activation of AKT and mTOR pathways *in vitro*. **(A)** Aurora-A-induced PTX- resistance is blocked by AKT inhibitor (Perifosine, 2.5 μM) in Aurora-A stable-expressing HEC-1B cells. **(B)** Aurora-A-induced PTX- resistance is blocked by mTOR inhibitor (RAD001, 200 nM) in Aurora-A stable-expressing HEC-1B cells. **(C)** Genetic knockdown using specific shRNA against either AKT or mTOR blocks effect of Aurora-A-induced PTX-resistance in Aurora-A stable-expressing HEC-1B cells. **(D,E)** Similar to **(A,B)**, Aurora-A-induced CIS- resistance is blocked by AKT inhibitor and mTOR inhibitor, respectively. **(F)** Similar to **(C)**, genetic knockdown using specific shRNA against either AKT or mTOR blocks effect of Aurora-A-induced CIS-resistance in HEC-1B cells. The concentrations of PTX and CIS were 100 nM and 64 μM, respectively. Data are expressed as means ± S.E.M., one-way ANOVA, *N* = 5, ***P < 0.001 and ns: not significant in **(A–F)**.

Next, we wonder whether Aurora-A overexpression confers chemoresistance via the AKT-mTOR signaling pathway. The role of AKT-mTOR pathway activity in this positive feedforward effect was investigated via the treatment with the AKT inhibitor Perifosine or mTOR inhibitor RAD001. In Aurora-A stable-expressing HEC-1B cells (wild-type PTEN cell line), the results showed that both of the two inhibitors blocked Aurora-A-induced PTX-resistance at 72 h treatment (*P* < 0.001) (Figures 4A,B). Similarly, shRNA-mediated AKT or mTOR knockdown prevented the effect of Aurora-A-induced chemoresistance (P < 0.001) (Figure 4C). Furthermore, similar results were observed in Aurora-A-induced CIS-resistance (Figures 4D–F). Therefore, blockade of AKT or mTOR pharmacologically or downregulated *via* shRNA prevented the Aurora-A-induced chemoresistance effect. Similar results were also observed in Aurora-A stable-expressing Ishikawa cell (mutant PTEN cell line) (Supplementary Figure 2). Taken together, Aurora-A recruits the AKT-mTOR pathway to induce PTX- and CIS-resistance in HEC-1B and Ishikawa EC cell lines.

### Expression of Aurora-A, pAKT and p4E-BP1 in Human EC Tissues

**Figure 5:**
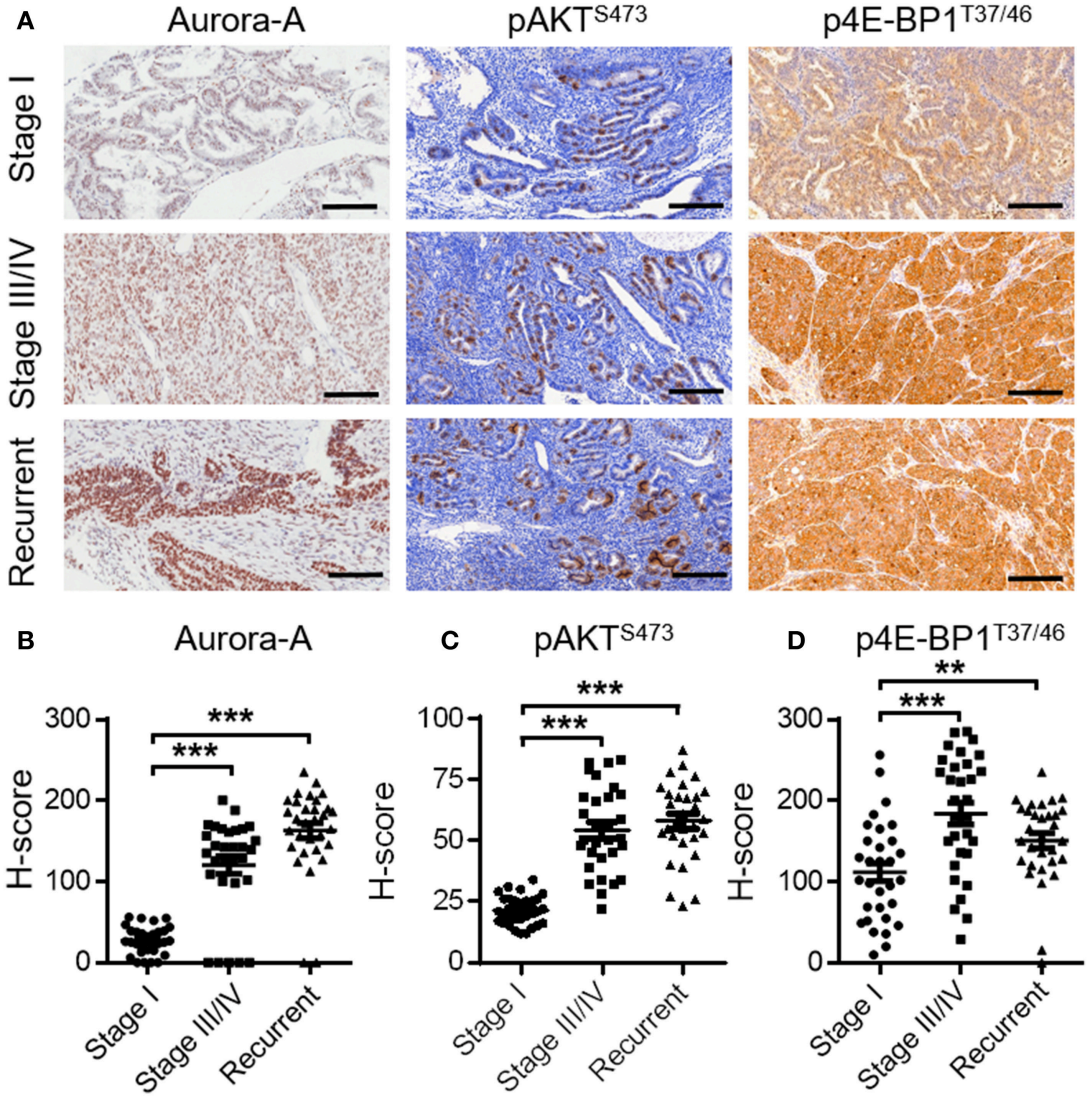
Expression of Aurora-A, pAKT and p4E-BP1 in human EC tissues**. (A)** Representative IHC staining showing Aurora-A, pAKT^S473^ and p4E-BP1^T37/46^ expression in tumor sections of EC patients (stage I, stage III/IV, and recurrent patients). Scale bar, 200 μm. **(B–D)** H-Scores of Aurora-A, pAKT^S473^, and p4E-BP1^T37/46^ levels were presented as a scatter diagram in **(A)**. Data are expressed as means ± S.E.M., Student's *t-test, N* = 30, **P < 0.01, ***P < 0.001.

To further illustrate the relationship between Aurora-A and AKT-mTOR pathway *in vivo*, we examined the expression level of Aurora-A and the phosphorylation status of AKT or 4E-BP1 in human EC tissues by IHC method. Considering Aurora-A expression is positively correlated with clinical stage and recurrence in EC patients, we selected 30 cases of early stage, 30 cases of advanced stage and 30 cases with recurrence for analyzing. Of note, the 30 cases with recurrence underwent PTX or CIS treatment. Results showed that the expression level of Aurora-A was upregulated in advanced and recurrent EC compared with the primary EC. Accordingly, the phosphorylation levels of AKT and 4E-BP1 also were increased significantly (Figure 5). Therefore, there was a statistically positive correlation between Aurora-A expression and phosphorylated AKT/4E-BP1 expression in EC tissues.

## Discussion

Over the past decades, Aurora-A has been studied in several human cancers, and Aurora-A has attracted a great deal of interest as a potential therapeutic target due to its overexpression in cancers (7). Aurora-A is an oncogene in mammary epithelium and gland (11, 12), whereas it functions as a tumor suppressor in neural stem cells (13), so Aurora-A functions differ depending on the cell type. Aurora-A has been reported in the gynecologic cancers, such as breast cancer (18), ovarian cancer (20, 24), and EC (22), but the underlying molecular mechanism of Aurora-A-mediated chemoresistance in EC is unclear. In the present study, we revealed that both of mRNA and protein up-regulation of Aurora-A frequently occur in EC and contribute to a poor prognosis. Furthermore, we demonstrate that overexpressed Aurora-A promotes cell proliferation and induces paclitaxel- and cisplatin-resistance in EC Ishikawa and HEC-1B cells, a result consistent with previous study (22). Our findings suggest that Aurora-A is an oncogene in EC and plays an important role in chemoresistance.

Chemotherapy therapy is a mainstay treatment option for advanced and recurrent EC, but chemoresistance remains a challenge for successful management of this malignancy (1). Thus, understanding the mechanisms of chemoresistance will be useful for targeted EC treatment. Deregulations in the apoptotic pathways (such as p53, Fas/FasL, Bcl-2 family proteins, inhibitor of apoptosis proteins) and survival pathways (PI3K/AKT/mTOR, MAPK) are considered as key pathways involved in the onset and maintenance of therapeutic resistance in EC (3), we identified that AKT/mTOR pathway was specifically activated by Aurora-A to enhances PTX and CIS chemosensitivity in EC cells. Using bioinformatics analysis in combination with pharmacological inhibition or shRNA-mediated knockdown, and subsequent cell viability assay, we systematically revealed that Aurora-A enhanced PTX and CIS chemosensitivity by up-regulation of the AKT/mTOR signaling pathway in EC Ishikawa and HEC-1B cell lines Accordingly, a synergetic relationship between Aurora-A expression and AKT/mTOR signaling was also clearly observed in EC tissues. AKT/mTOR signaling pathway has been involved in resistance to both targeted and cytotoxic therapy in multiple tumors and plays a crucial role in cell growth and survival, which justifies the desired target for pharmacological intervention (28). Now, AKT inhibitor MK2206, mTOR inhibitors Ridaforolimus, Everolimus, and Temsirolimus are undergoing a phase 2 trial for EC treatment (1). Importantly, Aurora-A inhibitor and chemotherapeutic agents as a targeted combination therapy for pancreatic cancer, head and neck squamous cell carcinoma and gastrointestinal adenocarcinomas have achieved promising results (29–31). Of particular note, Aurora-A is overexpressed in the EC patients who have a poor prognosis. Thus, inhibition both of Aurora-A and AKT/mTOR may represent a novel therapeutic approach for the chemo-resistant phenotype in EC patients.

Interestingly, IHC staining showed that Aurora-A was mainly located in the nucleus but not cytoplasm of EC tissues, a result consistent with previous study (22). This is very interesting, because Aurora-A is a kinase, and should be mainly located in the cytoplasm in normal tissues and cancer tissues. However, Aurora-A was highly expressed in the nuclear fraction of EC tissues, indicating that the nuclear localization of Aurora-A would be important during EC development, with cell-type specific functions. Of particular note, although kinase-dependent functions of Aurora-A are studied for several decades, kinase-independent functions are not yet fully understood. Emerging evidences indicate that Aurora-A performs functions independently of its kinase activity (32), for instance, recent study showed that Aurora-A interacts with heterogeneous nuclear ribonucleoprotein K (hnRNP K) in the nucleus and acts as a transcription factor in a complex that regulates *MYC* gene expression (33). Therefore, the functions of nuclear Aurora-A in EC remain an interesting question and needs to be explored in the further study.

In summary, our study demonstrate that high expression of Aurora-A is correlated with poor survival outcome for EC patients. Moreover, Aurora-A promotes cell proliferation and induces chemoresistance through activation of the AKT/mTOR pathway in EC. Further clinical studies are warranted to establish novel therapeutic strategies, such as using the Aurora-A inhibitor and either AKT or mTOR inhibitor in combination with conventional chemotherapy, to improve clinical benefit for EC patients with overexpression of Aurora-A.

## Author Contributions

JW, GY, and HZ designed the experiments and prepared the manuscript. JW, ZC, XX, JF, TL, KW, XK, and QY performed the experiments. HZ and GY conceived and supervised the project. All authors analyzed the data, read, and approved the final manuscript.

### Conflict of Interest Statement

The authors declare that the research was conducted in the absence of any commercial or financial relationships that could be construed as a potential conflict of interest.

## Abbreviations

ANOVA: Analysis of variance
BMI: Body mass index
CCK-8: Cell Counting Kit-8
CIS: cisplatin
EA: Endometrioid adenocarcinoma
EC: Endometrial cancer
GSEA: Gene Set Enrichment Analysis
IHC: Immunohistochemistry
PTX: paclitaxel
qRT-PCR: quantitative real-time polymerase chain reaction
S.E.M: Error of the mean.
