# Supplementary material for: Aurora-A Induces Chemoresistance Through Activation of the AKT/mTOR Pathway in Endometrial Cancer
Source: Front Oncol. 2019 May 22;9:422. doi: 10.3389/fonc.2019.00422 (PMC6540875; doi:10.3389/fonc.2019.00422)
Supplement: Supplementary file 1 [file Data_Sheet_1.docx]

**Supplementary Data**

**
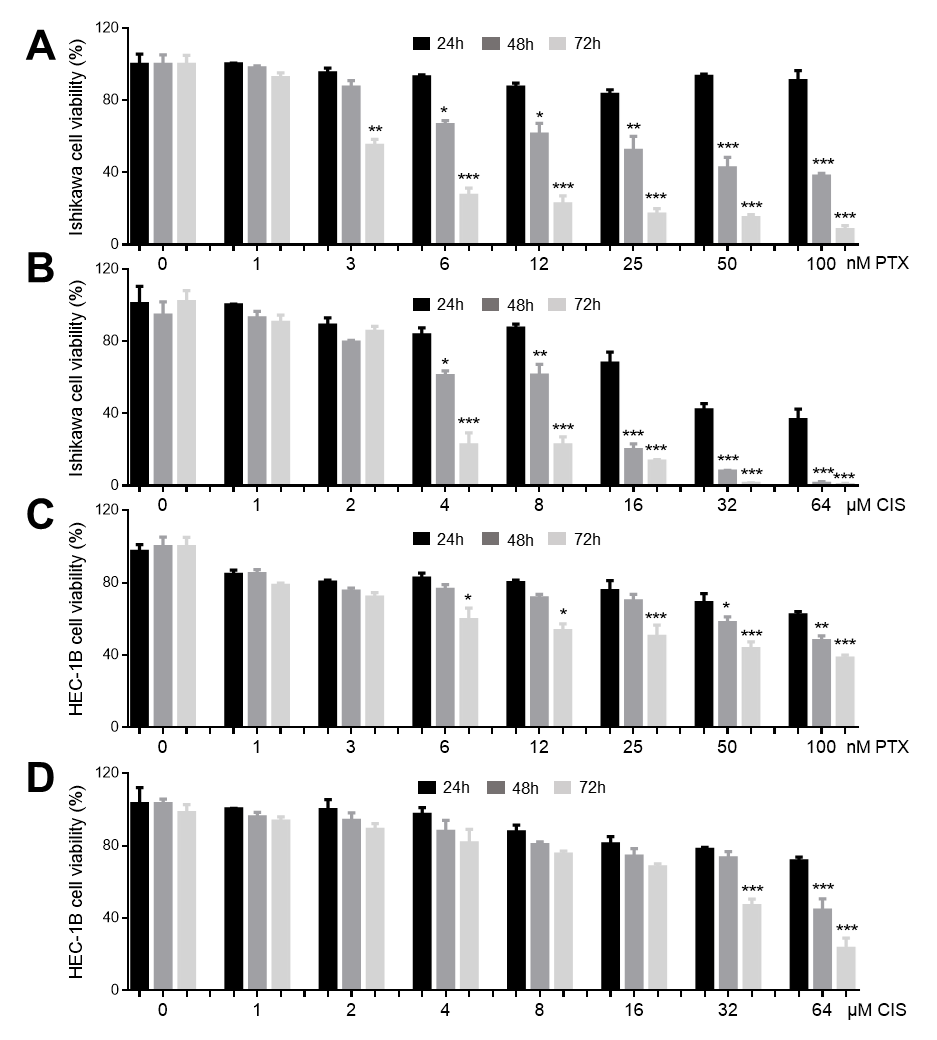
**

**Supplementary Figure 1.** PTX and CIS effects on Ishikawa and HEC-IB cell viability. Ishikawa and HEC-IB cells were incubated for 24, 48, and 72 h in culture medium supplemented without or with increasing concentrations of PTX and CIS, respectively. Cell viability was evaluated with three replicates each. **(A)**. Ishikawa cells treated with 0~100 nM PTX. **(B)** Ishikawa cells treated with 0~64 μM CIS. **(C)** HEC-IB cells treated with 0~100 nM PTX. **(D)** HEC-IB cells treated with 0~64 μM CIS. Data are expressed as means ± S.E.M., one-way ANOVA, N=3, ***P < 0.001, **P < 0.01 and *P < 0.05.

**
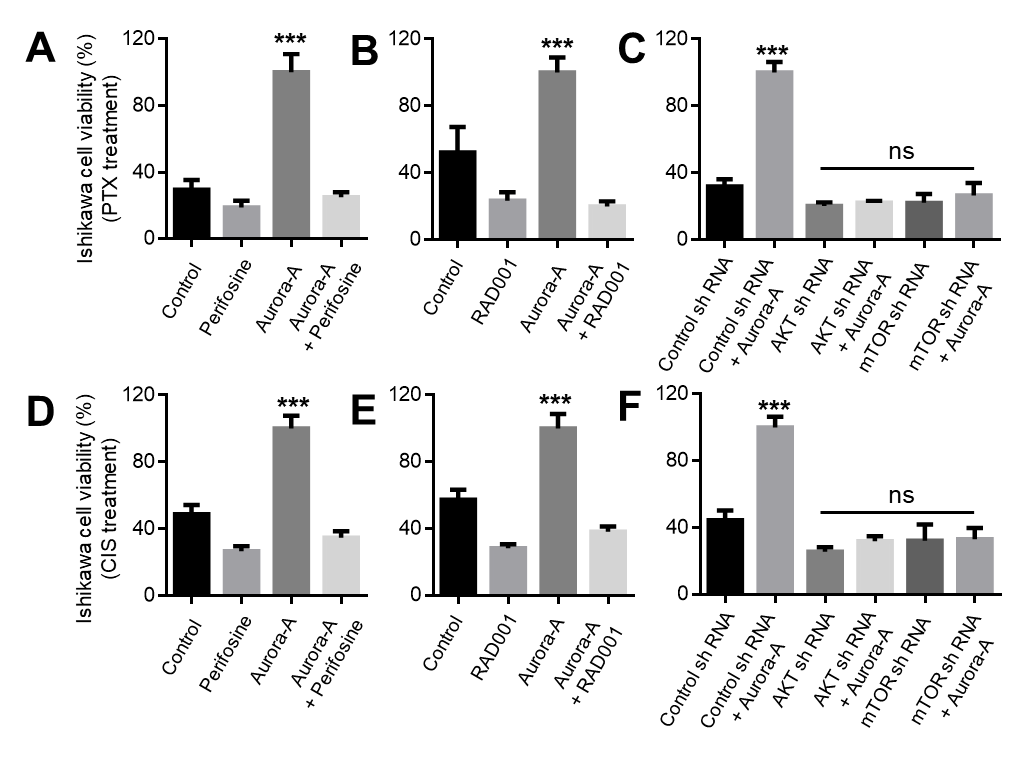
**

**Supplementary Figure 2. Aurora-A induces chemoresistance by activation of AKT and mTOR pathways in vitro. (A)** Aurora-A–induced PTX- resistance is blocked by AKT inhibitor (Perifosine, 2.5µM) in Ishikawa cells. **(B)** Aurora-A–induced PTX- resistance is blocked by mTOR inhibitor (RAD001, 200 nM) in Ishikawa cells. **(C)** Genetic knockdown using specific shRNA against either AKT or mTOR blocks effect of Aurora-A-induced PTX-resistance in Ishikawa cells. **(D-E)** Similar to (A-B), Aurora-A-induced CIS- resistance is blocked by AKT inhibitor and mTOR inhibitor, respectively. **(F)** Similar to (C), Genetic knockdown using specific shRNA against either AKT or mTOR blocks effect of Aurora-A–induced CIS- resistance in Ishikawa cells. The concentrations of PTX and CIS were 25 nM and 12 µM, respectively. Data are expressed as means ± S.E.M., one-way ANOVA, N=5, ***P < 0.001 and ns: not significant in (A-F).

**Supplementary Table 1**

Gene sets enrichment analysis in Aurora-A phenotype

| MSigDB collection | Gene set name | NES | NOM P-val | FDR q-val |
| --- | --- | --- | --- | --- |
| c6.all.v6.2.symbols.gmt | AKT_UP.V1_DN | -1.8655727 | 0.003976143 | 0.22745954 |
|  | RPS14_DN.V1_UP | -1.8626153 | 0.002109705 | 0.11628942 |
|  | MTOR_UP.V1_DN | -1.8182838 | 0.003976143 | 0.11461353 |
|  | ATF2_S_UP.V1_DN | -1.7766628 | 0.011428571 | 0.123074085 |
|  | ATF2_UP.V1_DN | -1.7698643 | 0.011811024 | 0.104228646 |
|  | STK33_UP | -1.7150114 | 0.006521739 | 0.13576981 |
|  | STK33_NOMO_UP | -1.7021928 | 0.012371134 | 0.13028547 |
|  | AKT_UP_MTOR_DN.V1_DN | -1.6533005 | 0.01875 | 0.16304304 |

NES: normalized enrichment score; NOM: nominal; FDR: false discovery rate. Gene sets with NOM P-val <0.005 and FDR q-val <0.075 are considered as significant.
